# The mitochondrial genome of *Dendroctonus rufipennis* (Kirby, 1837) (Coleoptera: Curculionidae: Scolytinae) and its phylogenetic analyses

**DOI:** 10.1080/23802359.2022.2161840

**Published:** 2023-01-13

**Authors:** Rui Meng, Hu Tian, Wei Lin, Yi-Xin Huang, Bo Cai

**Affiliations:** a Post-Entry Quarantine Station for Tropical Plant, Haikou Customs District P.R. China, Haikou, P.R. China; b Hainan Province Engineering Research Center for Quarantine, Prevention and Control of Exotic Pests, Haikou, P.R. China; c Caofeidian Customs District P.R. China, Tangshan, P.R. China; d Technical Center of Gongbei Customs District P.R. China, Zhuhai, P.R. China; e School of Ecology and Environment, Anhui Normal University, Wuhu, P.R. China

**Keywords:** Hylurgini, mitogenome, phylogenetic relationship

## Abstract

The mitogenome of *Dendroctonus rufipennis* is a circular molecule of 16,547 bp which consists of 13 protein-coding genes (PCGs), 22 transfer RNA genes, two ribosomal RNA genes, and a control region (GenBank accession no. NC_063906). All of 13 PCGs initiate with the standard start codon of ATN. Most PCGs used the typical stop codon ‘TAA’ or ‘TAG’, only *nad5* terminated with incomplete stop codon (TA). Phylogenetic analyses within the Scolytinae were performed based on nucleotide sequences of mitochondrial PCGs. The topology showed that *D. rufipenni*s, *D. valens*, and *Tomicus piniperda* formed a clade, and also clustered together with *Hylastes opacus*, *H. attenuatus*, *H. brunneus*, and *H. ater*. This indicated that there is a close genetic relationship between these species.

*Dendroctonus rufipennis* (Kirby, 1837) (Coleoptera: Curculionidae: Scolytinae) is one of the most destructive pests of spruce forests in North America. Under favorable conditions, outbreaks can develop and kill extensive forests over large areas (Werner et al. 1977). It is a quarantine pest in the list of entry plant quarantine pests of the People’s Republic of China and frequently intercepted in log quarantine at entry ports of China. To date, the mitogenome sequence of *D. rufipennis* remains unknown. Therefore, the mitochondrial genome of *D. rufipennis* was sequenced to provide more comprehensive data for this species.

The species is mainly identified by the following characteristics (Figures 1 and 2): adults are blackish-brown to black with reddish-brown or black elytra; range in length from 3.4 to 5.0 mm long and about 3 mm wide; frons without deep median groove between eyes, punctures usually obscure in central area; epistomal process narrower, distance between eyes about three times than its basal width, with moderately oblique lateral margins (anterior angles at 55°); elytra are 2.5 times the length of the pronotum; declivital striae at most weakly impressed, declivital striae 2 as wide or wider than 1 or 3, strial punctures on elytral declivity no more than two times as large as interstrial punctures (Wood 1982).

**Figure 1.**
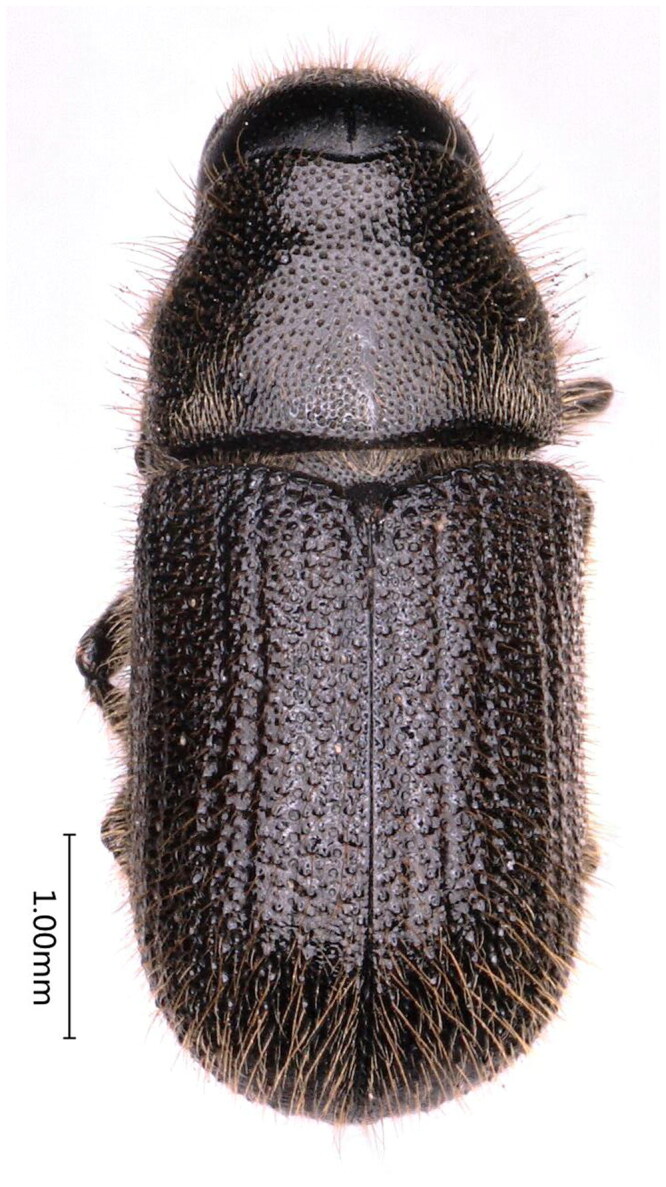
Dorsal view of *D. rufipennis*, photographed by Hu Tian.

**Figure 2.**
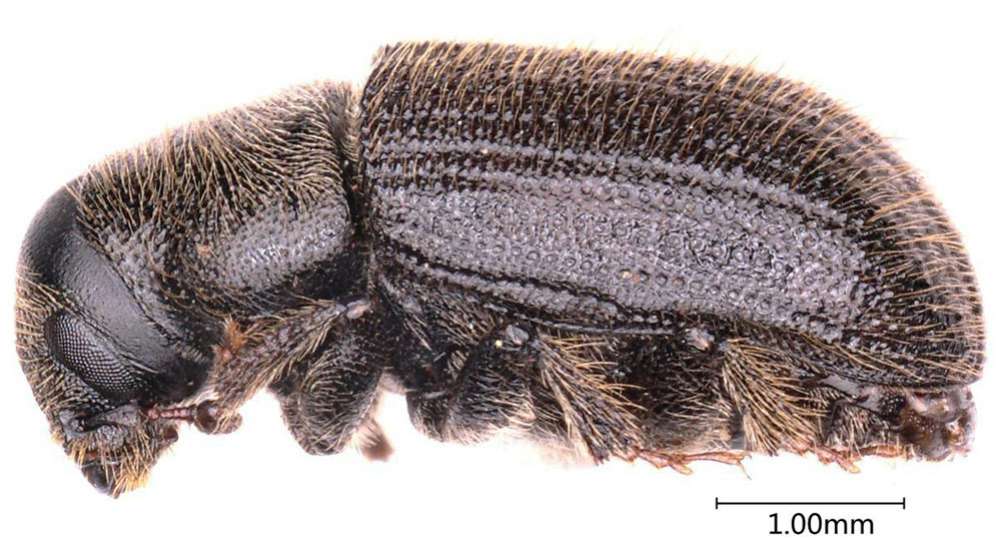
Lateral view of *D. rufipennis*, photographed by Hu Tian.

In this study, adult samples of *D. rufipennis* were intercepted in the Spruce logs import from British Columbia of Canada at Caofeidian Port (39°6′17″N, 118°30′29″E) in March 2020. The specimens were deposited at −20 °C in herbarium of Post-Entry Quarantine Station for Tropical Plant, Haikou Customs District P.R. China (specimen accession number: IN06130107-0001-0003, DNA voucher R109) (URL, Meng Rui, huamei0391@163.com). The mitogenome was sequenced using the Illumina HiSeq X TEN Sequencing System 2500 platform with 150 bp paired-end reads. The assembly method used here adopts the method of Meng et al. (2019), and uses the Mitoz software package to conduct the assembly with default parameters. The annotations were mainly compared with the existing mitochondrial genomes of related species, and the annotation results were confirmed and modified by MITOS online tool (Bernt et al. 2013).

The nearly complete mitogenome sequence of *D. rufipennis* with a length of 16,937 bp (GenBank accession number NC_063906), contained 13 protein-coding genes (PCGs), 22 tRNA genes, two rRNA genes, and a control region (Figure 3). There are 23 genes encoded on the heavy strand, while the remaining 14 genes encoded on the light strand. The nucleotide composition of the sequenced mitogenome sequence was biased toward AT 73.7%, and the total base composition was 40% A, 33.7% T, 10.6% G, and 15.6% C. The length of 13 PCGs was 11,132 bp, all PCGs started with ATN, ATA for *atp8*, *cox3*, *nad3*, *cytb*, and *nad1*; ATG for *atp6*, *nad4*, and *nad4L*; ATT for *cox1*, *cox2*, *nad5*, and *nad6*, ATC for *nad2*. Except for *nad5* used TA as the stop codon, the remaining 12 PCGs were inferred to terminate with the complete stop codon (TAA or TAG). Twenty-two tRNA genes were identified and ranged in length from 61 bp to 70 bp. The large ribosomal gene (rrnL) is 1347 bp in length with an AT content of 80.2%, which was found between trnL1 and trnV. The small ribosomal gene (rrnS) is 809 bp with an AT content of 77.5%, and positioned between trnV and the control region. The major non-coding region between rrnS and trnI corresponding to control region, with A + T contents are 83%, which is obviously higher than the A + T content of the entire mitogenome. A conserved overlapping sequence motif ‘ATGATAG’ between ATP8 and ATP6 is present, this motif was also found in other mitogenomes from Scolytinae (Lv et al. 2017).

**Figure 3.**
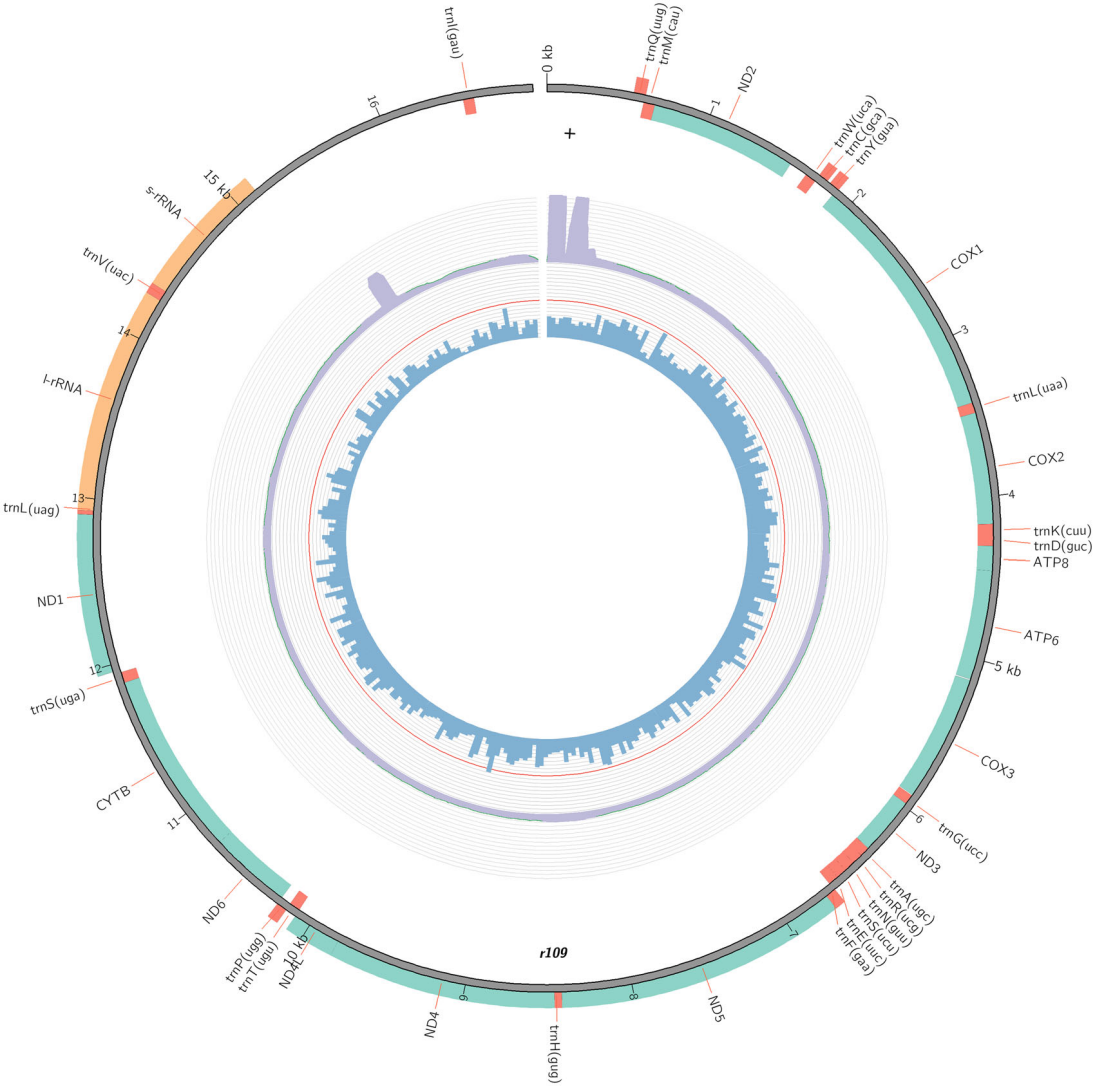
Circular map of the mitogenome of *D. rufipennis.*

To validate the phylogenetic position of *D. rufipennis* based on mitogenome, we selected mitogenomes of 45 species which represent all Scolytinae 23 genera in 13 tribes currently deposited in the GenBank (Figure 4). Two species respectively from Curculioninae and Cryptorhynchinae with close phylogenetic relationship to Scolytinae were selected as outgroup (Zhang et al. 2022). Nucleotide sequences were aligned, concatenated, and partitioned in Phylosuite (Katoh and Standley 2013; Zhang et al. 2020). Best partitioning scheme and evolutionary models for 13 pre-defined partitions were selected using PartitionFinder2 (Lanfear et al. 2017), with greedy algorithm and AIC criterion. The maximum-likelihood (ML) tree based on 13 PCGs was reconstructed in Phylosuite using partition model with 5000 bootstrap replicates (Nguyen et al. 2015; Zhang et al. 2020). The results showed that *D. rufipennis*, *D. valens*, and *Tomicus piniperda* in the Hylurgini formed a clade. The Hylurgini with Hylastini including *Hylastes opacus*, *H. attenuatus*, *H. brunneus*, and *H. ater* are clustered into one big branch. It indicated a close genetic relationship between these two tribes, this result was similar to the previous study (Johnson et al. 2018; Pistone et al. 2018). The independence of Trypophloeini is well supported in this study, in which tribal status has just been recovered in recent morphological study (Johnson et al. 2020). In conclusion, although this study provided important information for the phylogeny and evolution analysis in Scolytinae, more mt-genome sequences from all the 29 currently recognized tribes (Hulcr et al. 2015; Johnson et al. 2020) are also required in further studies to resolve the phylogenetic relationships within the whole subfamily.

**Figure 4.**
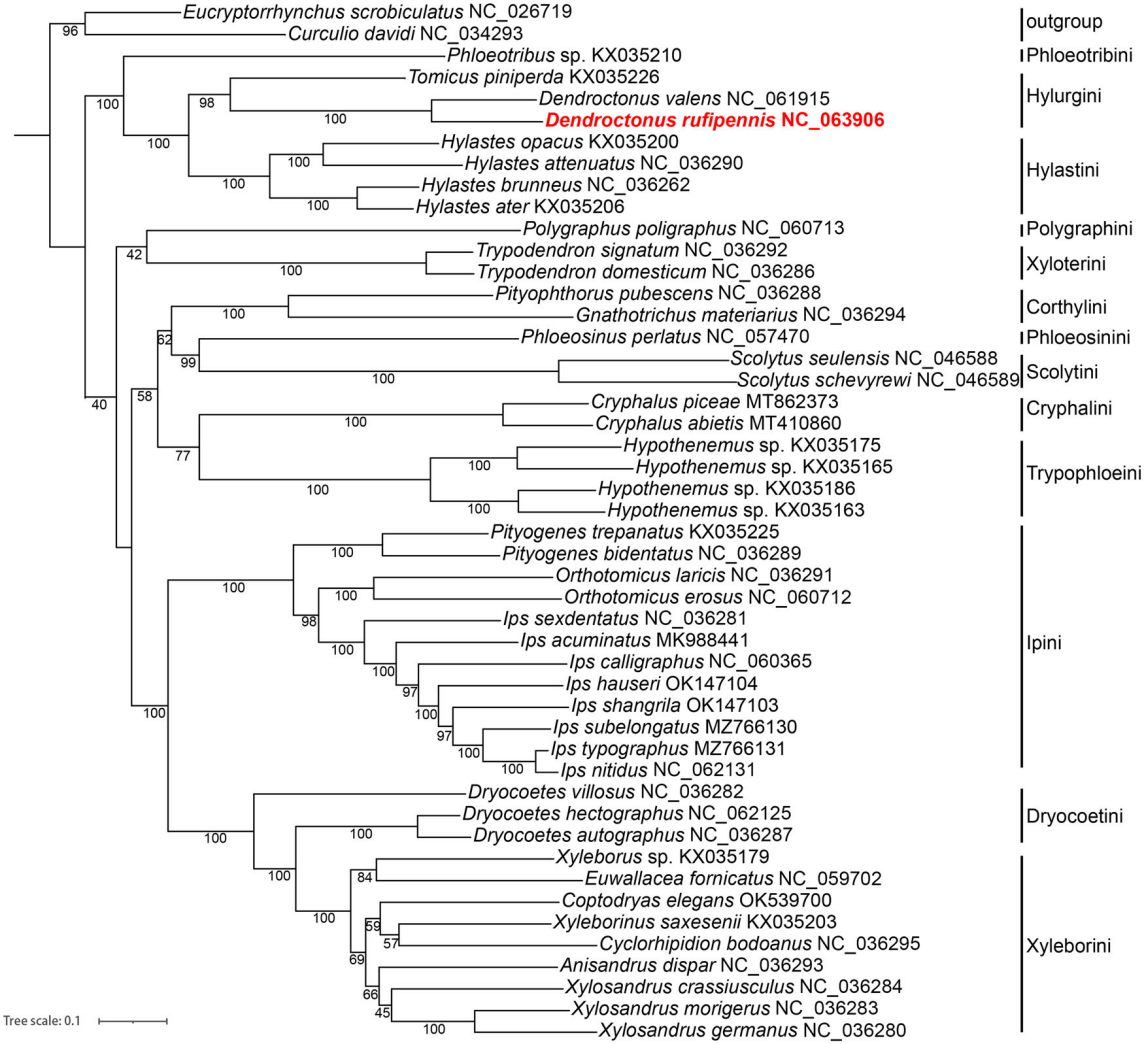
Phylogenetic tree showing the relationship between *D. rufipennis* and other Scolytinae species based on ML methods. Numbers on branches are Bootstrap values. *Eucryptorrhynchus scrobiculatus* NC_026719 (Liu et al. 2016); *Curculio davidi* NC_034293 (Xu et al. 2017); *Dendroctonus valens* NC_061915 (Zhang et al. 2022); *Ips calligraphus* NC_060365 (Xu et al. 2021); *Polygraphus poligraphus* NC_060713, *Orthotomicus erosus* NC_060712, *I. subelongatus* MZ766130, *I. typographus* MZ766131, *I. nitidus* NC_062131, *Dryocoetes hectographus* NC_062125 (Du et al. 2021); *I. hauseri* OK147104, *I. shangrila* OK147103 (Du et al. 2022); *Euwallacea fornicatus* NC_059702 (Wang et al. 2020); *Coptodryas elegans* OK539700 (Guo et al. 2022); *Phloeotribus* sp. KX035210, *Tomicus piniperda* KX035226, *Hylastes opacus* KX035200, *H. attenuatus* NC_036290, *H. brunneus* NC_036262, *H. ater* KX035206, *Trypodendron signatum* NC_036292, *T. domesticum* NC_036286, *Pityophthorus pubescens* NC_036288, *Gnathotrichus materiarius* NC_036294, *Phloeosinus perlatus* NC_057470, *Scolytus seulensis* NC_046588, *S. schevyrewi* NC_046589, *Cryphalus piceae* MT862373, *C. abietis* MT410860, *Hypothenemus* sp. KX035175, KX035165, KX035186, KX035163, *Pityogenes trepanatus* KX035225, *P. bidentatus* NC_036289, *Orthotomicus laricis* NC_036291, *I. sexdentatus* NC_036281, *I. acuminatus* MK988441, *Dryocoetes villosus* NC036282, *D. autographus* NC_036287, *Xyleborus* sp. KX035179, *Xyleborinus saxesenii* KX035203, *Cyclorhipidion bodoanus* NC_036295, *Anisandrus dispar* NC_036293, *Xylosandrus crassiusculus* NC_036284, *X. morigerus* NC_036283, *X. germanus* NC_036280 (unpublished).

## Data Availability

The data that support the findings of this study are openly available in GenBank of NCBI at https://www.ncbi.nlm.nih.gov/genbank/, reference number NC_063906. The associated BioProject, Bio-Sample numbers, and SRA are PRJNA874168, SAMN30527013, and SRR21245983, respectively.
